# Focally Enlarged Perivascular Spaces in Pediatric and Adolescent Patients with Polymicrogyria—an MRI Study

**DOI:** 10.1007/s00062-024-01457-5

**Published:** 2024-09-13

**Authors:** Maximilian Rauch, Karsten Lachner, Lea Frickel, Monika Lauer, Simon Jonas Adenauer, Elisabeth Neuhaus, Elke Hattingen, Luciana Porto

**Affiliations:** 1 Institute for Neuroradiology, Johann Wolfgang Goethe-University, Theodor Stern Kai 7, 60590 Frankfurt am Main, Germany; 2 Department of Radiology, Helios Klinikum Bonn/Rhein-Sieg, Von-Hompesch-Straße 1, 53123 Bonn, Germany

**Keywords:** Polymicrogyria, Malformations of cortical development, Magnetic resonance imaging, Neuroimaging

## Abstract

**Purpose:**

Polymicrogyria (PMG) is a cortical malformation frequently associated with epilepsy. Our aim was to investigate the frequency and conspicuity of enlarged perivascular spaces (EPVS) underneath dysplastic cortex as a potentially underrecognized feature of PMG in pediatric and adolescent patients undergoing clinical magnetic resonance imaging (MRI).

**Methods:**

We analyzed data from 28 pediatric and adolescent patients with PMG and a matched control group, ranging in age from 2 days to 21 years, who underwent MRI at 1.5T or 3T. T2-weighted MR images were examined for the presence of EPVS underneath the dysplastic cortex. The quantity of EPVS was graded from 0 to 4 (0: none, 1: < 10, 2: 11–20, 3: 21–40, 4: > 40 EPVS). We then compared the presence and quantity of EPVS to the matched controls in terms of total EPVS scores, and EPVS scores underneath the dysplastsic cortex depending on the age groups, the localization of PMG, and the MRI field strength.

**Results:**

In 23/28 (82%) PMG patients, EPVS spatially related to the dysplastic cortex were identified. EPVS scores were significantly higher in PMG patients compared to controls, independent from age or PMG location. No significant differences were observed in EPVS scores in patients examined at 1.5T compared to those examined at 3T.

**Conclusion:**

EPVS underneath the dysplastic cortex were identified in 82% of patients. EPVS may serve as an important clue for PMG and a marker for cortical malformation.

## Introduction

Polymicrogyria (PMG) is a malformation of the cerebral cortex that results from abnormal neuronal migration and cortical disorganization [1, 2]. It is frequently associated with epilepsy. The morphologic hallmark of PMG is an excessive number of abnormally small gyri that are separated by shallow sulci [3]. A characteristic of PMG appearance is irregularity at both the cortical surface and the corticomedullary junction [2].

PMG is a heterogeneous malformation with various clinical and imaging features, pathological findings, and etiologies [4–7]. Although PMG may occur in isolation, it may be associated with other brain or body malformations. Numerous genetic and nongenetic causes of PMG have been identified, such as congenital cytomegalovirus infection and in utero ischemia [8]. PMG is typically diagnosed using magnetic resonance imaging (MRI) without pathological confirmation, as most patients will not undergo surgical treatment. Therefore, diagnosis and severity have been qualitatively judged by visual inspection of imaging features.

Focal EPVS have been described as an epiphenomenon in various congenital, vascular, inflammatory, and degenerative cerebral diseases, although their significance is a matter of debate [9–13]. The presence of focally enlarged perivascular spaces (EPVS) in the white matter close to the polymicrogyric cortex has already been reported [14, 15], but the prevalence of these PMG-associated EPVS on clinical MRI examinations has not yet been systematically analyzed.

In this study, we aimed to consolidate our observation that focally EPVS underneath the dysplastic polymicrogyric cortex are a frequent and potentially underrecognized feature in pediatric and adolescent PMG-patients undergoing clinical 1.5T and 3T MRI.

## Material and Methods

### Data Acquisition

MRI studies were performed in neuroradiologic departments of two health care centers. The institutional review boards of the two participating centers approved this retrospective study. Informed consent was waived.

We reviewed the radiological databases of both institutions for patients that underwent MRI from 2009 to 2022 using the search term ‘polymicrogyria’, yielding a collective of 179 patients.

MRIs from patients with other diagnoses than PMG (*n* = 126), patients > 21 years (*n* = 20) and examinations with poor quality (*n* = 5) were excluded.

Data of the remaining 28 patients were used for further analysis. Imaging was performed on 1.5T (*n* = 14) and 3T (*n* = 14) MR scanners. Clinical data were collected and analyzed.

We further reviewed the radiological databases to create a control group of patients with normal brain MRIs, matching age, gender and MRI field strengths. Patients in whom vascular disease, infection, inflammation, and seizures were listed as indications for MRI were excluded as controls to avoid a possible bias regarding the occurrence of EPVS. A patient-to-control ratio of 1:2 (28:56) was applied.

According to MRI findings in white matter maturation [16], patients were categorized into the following age groups: 0–9 months, 10–24 months, > 24 months.

The MRI protocols in PMG patients and control subjects included T1, T2, FLAIR, 3D T2, T1 MPRAGE and T1 MP2RAGE sequences. An overview of these is provided in Table 1.

**Table 1:** MRI sequence parameters in PMG patients (*n* = 28) and controls (*n* = 56) and number of subjects in which they were available

Sequence type/weighting	*n* PMG	*n* control	Primary acquisition plane	TR [ms]	TE [ms]	FA	Matrix	FOV [mm^2^]	Slice/section thickness [mm]	Spacing [mm]
T1‑w spin echo	28	56	Axial	200–620	3–12	90°	256 × 256–512 × 512	230 × 230	4–5	5–6
T2‑w turbo spin echo	28	56	Axial	3300–4200	80	90°	512 × 512	230 × 230	4–5	5–6
2D FLAIR	22	40	Axial	6000–12,000	120–140	90°	256 × 256–512 × 512	230 × 230–256 × 256	2–5	2–6
3D T2	4	10	Sagittal	1500	120	Variable	256 × 256–512 × 512	230 × 230–256 × 256	1.1	0.6
3D FLAIR	6	16	Sagittal	4800	300	90°	256 × 256–576 × 576	240 × 240–250 × 250	1.1	0.6
3D T1 MPRAGE	19	40	Sagittal	8–15	4	8°	256 × 256	256 × 256	1	1
3D T1 MP2RAGE	2	5	Sagittal	5000	2.8	5°; 6°	192 × 192	240 × 240	1	1

*FA* flip angle; *FOV* field of view; *TE* time echo; *TR* time repetition; *w* weighted

In patients with PMG, the diagnosis was initially confirmed in each case by a senior pediatric neuroradiologist (20 years of experience) who considered all available sequences. A diagnosis of PMG was made if the affected cortex met the following criteria [5]: (1) irregular cortical surface, (2) thickened or overfolded appearing cortex, (3) ‘stippling’ or irregularity at the grey-white matter interface. We classified PMG according to localization (generalized, frontal, perisylvian, parasagittal, parietal, others [5]) and lateralization (right, left, bihemispheric). In the controls, normal findings were confirmed by the same neuroradiologist. Scans of the PMG group and the controls were then blinded.

### Image Analysis

Axial T2-weighted MR images (Table 1) were then reviewed in consensus by two readers (4 and 5 years of experience in pediatric neuroradiology) on a radiology workstation (Centricity 7.0, GE Healthcare, Chicago, USA).

The presence or absence of EPVS in each location was graded using the following scale [17, 18]: 0 = no EPVS, 1 = < 10 EPVS, 2 = 11 to 20 EPVS, 3 = 21 to 40 EPVS, and 4 = > 40 EPVS.

EPVS were defined as small, sharply delineated structures of T2 hyperintense cerebrospinal fluid intensity that followed the orientation of the perforating vessels perpendicular to the brain surface. In addition, the structures had to be irregular in shape to be distinguished from normal, non-enlarged perivascular spaces. Lesions > 3 mm in size and spheroidal in shape were classified as lacunae and not as EPVS.

### Statistical Analysis

Statistical analysis was performed using Microsoft Excel version 16.78 (Microsoft, Redmond, WA, USA) and GraphPad Prism (version 6.0, GraphPad Software, Boston MA, USA). Data distribution was ascertained using the D’Agostino-Pearson omnibus normality test. In order to analyze the non-normally distributed ordinal scaled data, non-parametric tests were employed. In order to ascertain whether there were any significant differences in the EPVS scores observed in the various age groups and PMG subtypes, a comparison was made between the EPVS scores recorded underneath the polymicrogyric cortex and those of the corresponding localizations in the control group. The Kruskal-Wallis test was utilized for this purpose. To compare EPVS scores for patients who underwent 1.5T MRI with those who underwent 3T MRI, the Mann-Whitney U test was employed.

A *p*-value < 0.05 was considered as statistically significant.

## Results

We analyzed MRIs of 16 male and 12 female PMG patients and their controls matched for age, sex and MRI field strengths. Demographics of PMG patients are shown in Table 2. Indications for MRI in the PMG group included seizures (47%), suspected congenital brain malformation (32%), intellectual disability (14%) and congenital cytomegalovirus (CMV) infection (7%) (Table 3). The indications for the MRIs of the controls that were found to be unremarkable included headache (25%), suspected intracranial trauma (23%), strabismus (14%), depression (11%), cephalohematoma (7%), visual impairment (7%), vertigo (5%), nystagmus (4%), staging for extracranial malignancy (2%), and suspected cerebral manifestations of thalassemia (2%).

**Table 2:** Demographics of patients with PMG

PMG Patients	*n*
Male	16
Female	12
*MRI*	*n*
1.5T	14
3T	14
**Age**	2 days–21 years (mean 5.7 years)
*Age groups*	*n*
0–9 months	8
10–24 months	2
> 24 months	17

**Table 3:** Indication for performing MRI in patients with PMG

Age	Congenital cytomegalovirus (CMV) infection	Seizures	Intellectual disability	Suspected congenital brain malformation
0–9 months (*n*)	2	3	–	4
10–24 months (*n*)	–	1	–	1
> 24 months (*n*)	–	9	4	4
Total	2	13	4	9

Table 4 presents the locations of PMG. Most patients had generalized PMG, followed by frontal PMG. In generalized PMG, the entire cerebral cortex was completely or almost entirely affected, with no regions with particularly pronounced involvement. Perisylvian PMG was unilateral in 5/8 cases, in all cases the insula was involved and to varying degrees the cortex of the adjacent temporal, frontal and parietal operculum. In the patient with parasagittal PMG, the dysplastic cortex was maximally pronounced in the parietal portion of the cingulate gyri and in the adjacent parietal lobes.

**Table 4:** Localizations and patterns of PMG

Localization	Subtype	*n*
Generalized	–	9
Frontal	–	7
Perisylvian	–	8
	Unilateral	5
	Bilateral symmetric	1
	Bilateral asymmetric	2
Parasagittal	–	1
Parietal	–	2
Other	–	1 (temporal)

Regarding associated non-cortical malformations, we found periventricular grey matter heterotopia in three patients (frontal *n* = 2, temporal *n* = 1).

**Fig. 1:**
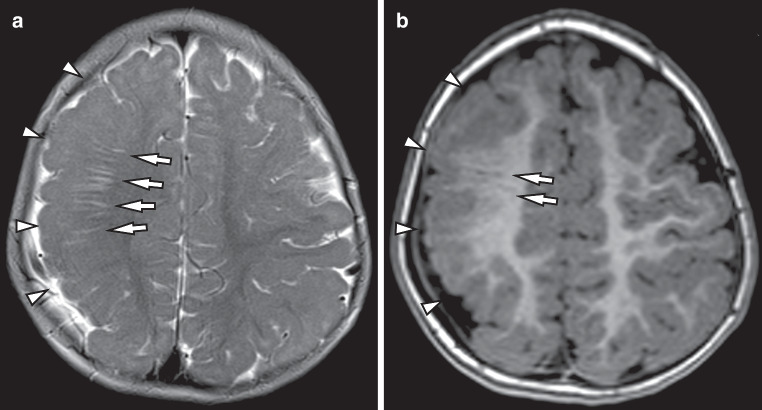
Enlarged perivascular spaces (*arrows*) underneath polymicrogyric cortex (*arrowheads*) in a 4 year old female patient with frontal and parietal lobar polymicrogyria (*arrowheads*). **a** Axial T2 weighted MR image. **b** Axial T1 weighted MPRAGE (magnetization prepared rapid acquisition with gradient echoes) image

Total EPVS scores were found to be significantly higher in PMG patients compared to controls (*p* < 0.0001). 23/28 (82%) PMG patients exhibited a greater number of EPVS underneath the dysplastic cortex compared to the white matter in the same location underneath normal cortex in controls (Fig. 1). The remaining five patients, who did not differ from the controls regarding quantity of EPVS, had frontal PMG in two cases and generalized PMG in three cases.

The EPVS scores were non-normally distributed in both the PMG and control groups. Statistic results for the EPVS scores are summarized in Table 5.

**Table 5:** Statistics for the enlarged perivascular space (EPVS) scores

	EPVS scores					*p*-value
	min	Median	Max	Mean	SD	
PMG (total)	0	2	4	*1.896*	*1.422*	< 0.0001
Control (total)	0	0	1	*0.1523*	*0.019*	
*Age groups*						
Underneath PMG: 0–9mo	0	1	4	*1.176*	*0.183*	< 0.0001
Control: 0–9 mo	0	0	0	*0*	*0*	
Underneath PMG: 10 mo–24 mo	3	4	4	*3.667*	*0.500*	< 0.0001
Control: 10 mo–24 mo	0	0	1	*0.111*	*0.111*	
Underneath PMG: > 24 mo	2	3	4	*2.620*	*1.144*	< 0.0001
Control: > 24 mo	0	0	1	*0.290*	*0.456*	
*PMG subtypes*						
Generalized PMG	0	2	4	*2.060*	*1.391*	< 0.0001
Control	0	0	1	*0.205*	*0.046*	
Frontal PMG	0	2	4	*1.636*	*0.491*	0.0313
Control frontal	0	0	0	*0*	*0*	
Perisylvian PMG	0	2	4	*2.844*	*1.224*	< 0.0001
Control perisylvian	0	0	1	*0.2439*	*0.068*	
Parietal PMG	4	4	4	*4*	*0*	–
Control parietal	0	0	0	*0*	*0*	
Parasagittal PMG	2	–	–	–	–	–
Control parasagittal	0	–	–	–	–	
Temporal PMG	4	–	–	–	–	–
Control temporal	0	–	–	–	–	
*MRI field strengths*						
Underneath PMG 1.5T	0	2	4	*2.058*	*1.409*	< 0.0001
Control 1.5T	0	0	1	*0.1279*	*0.036*	
Underneath PMG 3T	0	3	4	*2.397*	*1.362*	< 0.0001
Control 3T	0	0	1	*0.2192*	*0.049*	

*PMG* polymicrogyria; *min.* minimum; *max.* maximum, *SD* standard deviation; *mo* months; *y* years

Regardless of the age, there were significant differences regarding the quantity of EPVS within each age group and the corresponding controls. No significant differences were found in the frequency and conspicuity of EPVS in different locations of PMG. Furthermore, no significant differences were observed in EPVS scores in patients examined at 1.5T compared to those examined at 3T.

## Discussion

Perivascular spaces, also known as Virchow-Robin spaces are pial-lined fluid-filled tubular structures that surround arterial and venous walls as they course from the subarachnoid space into the brain parenchyma [19]. These spaces been shown to directly communicate with the subpial space rather than the subarachnoid space [19]. The function of perivascular spaces is largely unknown. Recent studies suggest that they may play a role in the so called glymphatic system, a brain-wide drainage system that serves immunologic functions by exchanging interstitial and cerebrospinal fluid, thereby disposing of potentially neurotoxic waste products such as β‑amyloid [20].

Since the introduction of high-field MRI, perivascular spaces have been increasingly identified, ranging up to 100% of all individuals undergoing MRI [21, 22].

There is no universal definition of when perivascular spaces are considered as enlarged. To differentiate normal perivascular spaces from EPVS, we used a common definition according to which EPVS are classified on the basis of their shape in which irregular widened perivascular spaces are considered enlarged, whereas regular linear perivascular spaces are considered not enlarged [18, 22, 23].

In healthy individuals, the prevalence of EPVS has been shown to range from 1.6 to 3% [22, 24]. New interest in perivascular spaces emerged as recent studies, particularly using 7T ultra high-field MRI, have highlighted the potential role of normal and enlarged perivascular spaces in various disorders, including epilepsy, vascular disease, neurodegenerative disorders, and inflammatory disease [15, 25–28]. Additionally, EPVS have been studied as potential biomarkers in epilepsy [15], cerebral small vessel disease [25], and neurodegeneration [27, 29].

In patients with epilepsy, it has been demonstrated that the region showing the greatest asymmetry of perivascular spaces is often the same region that contains the suspected seizure onset zone [15].

However, there is no clear explanation for how perivascular spaces become enlarged. Some possible causes that have been discussed include perivascular myelin loss, increased arterial wall permeability, fibrosis leading to occlusion of lymphatic drainage, and ex-vacuo dilatation resulting from brain atrophy [19]. Despite ongoing research, the origin and significance of these findings have not been fully clarified.

PMG is associated with white matter abnormalities and disorganization in proximity but also extending beyond the affected cortex [30, 31]. Therefore, EPVS underneath polymicrogyric cortex may be an accompanying feature resulting from disorganized neuronal migration, associated white matter hypoplasia and ex-vacuo dilatation of perivascular spaces.

PMG is known to change the appearance of the affected cortex from thin towards thick as a consequence of ongoing myelination [32]. Although maturation may influence the appearance and occurrence of EPVS, we did not detect any significant difference between age groups in the detectability of enlarged perivascular spaces in different sequences.

A study of 328 PMG patients found that 13% of patients had prominent perivascular spaces, with a significant correlation to frontal PMG [5]. Another study reported EPVS in 27% of patients in the proximity of the polymicrogyric cortex [14]. In our study, the frequency of EPVS in a pediatric and adolescent collective was as high as 82%. However, this may be attributed to the relatively high spatial resolution of the MR sequences and increased awareness of this finding. We used non-fluid-attenuated T2-weighted MR images to visualize EPVS as these clearly show perivascular spaces that are isointense to cerebrospinal fluid. Studies showed that a better visualization of EPVS thereby can be achieved through thinner sections and heavier T2-weighting [24, 33].

Our investigation has several limitations. We studied a collective with heterogenous location of PMG and the varying ages of the patients. However, PMG is a rare malformation that can be diagnosed from newborns to late adulthood [5]. PMG has high degree of heterogeneity in which its etiology encompasses genetic, infectious, metabolic and vascular factors, all of which may exert an influence on the development and occurrence of EPVS [34, 35]. In regard to localization, a unilateral, deeply infolded, heterotopic gray matter brain malformation with areas of PMG surrounded by an otherwise normal-appearing brain has been postulated to be caused by an vascular insult during the early prenatal period [34].

Additionally, the study’s retrospective design and the differences in MRI scanner field strengths and varying scan protocols are also limitations. As a consequence of its retrospective design and the differing questions that led to MRI, thin-slice 3D sequences were not available for all subjects. This may limit the relevance of our results in these instances, as the identification of PMG on thicker MRI sections may be impeded. Regarding the assessment of EPVS, it would furthermore be advantageous for future prospective studies to employ high-field MRI up to 7T and thin slice 3D T2-weighted sequences to optimize the delineation of perivascular spaces [36, 37].

In this retrospective study, a ratio of 1:2 between patients and controls was selected due to the limited availability of unremarkable MRI examinations as controls, particularly in young children. It would be beneficial for future studies investigating EPVS with MRI to aim for higher ratios in order to increase statistical power.

Moreover, although the two raters were initially blinded to the cases, they were aware of the possible presence of PMG, which could have led to bias. In potential future MRI studies addressing EPVS in the context of cortical disorders, the cortex could be removed through image post-processing and segmentation prior to evaluation [38, 39], thereby allowing the investigators to focus on the white matter.

The diagnosis of cortical malformations by MRI between the 10th month and the 2nd year of life is subject to challenge due to a lack of diagnostic certainity during this period. This phenomenon is attributed to the process of progressive myelination [1]. In most MRI sequences, the contrast between the cortex and the white matter is only slight, which can lead to uncertainty in the diagnosis of subtle cortical malformations at this age. The MP2RAGE sequence (magnetization-prepared 2 rapid acquisition gradient echoes), which is a volumetric T1-weighted sequence, represents a potential avenue for improvement [40]. This sequence has a favorable contrast-to-noise ratio and enables enhanced delineation of the interface between gray and white matter. Therefore, it is better suited for the detection of cortical malformations [41]. However, despite the use of this sequence, the small sample size in our study may still lead to uncertainty in the interpretation of the results in the age group from 10–24 months.

A criticism of MRI studies on congenital cerebral malformations is that findings are often not histopathologically confirmed [5]. In PMG, epilepsy surgery is rarely performed, so diagnosis relies solely on imaging. Although MRI may not have the resolution to delineate histologic details of the malformed cortex, radio-pathologic studies in PMG have shown a good correlation between MRI and pathologic findings [42].

We excluded adult patients from our study because EPVS in these individuals may be associated with other pathologies that are more likely than those suspected in PMG, such as small vessel disease [18, 43]. In this context, we classified lesions > 3 mm as EPVS as lacunae, although these are uncommon in pediatric and adolescent patients [44].

In conclusion, enlarged EPVS were frequently found in pediatric and adolescent patients with PMG on clinical 1.5T and 3.0T MRI, regardless of the location of PMG. The detection of focal EPVS should therefore draw attention to the overlying cerebral cortex and may help to avoid missing PMG, especially in patients with epilepsy. EPVS may be a useful adjunct for MRI-based diagnosis of PMG if identification with classic imaging criteria is ambiguous.
